# Hospital preparedness for mass critical care during SARS-CoV-2 pandemic

**DOI:** 10.1186/s13054-020-03104-0

**Published:** 2020-06-30

**Authors:** Thomas Wurmb, Katja Scholtes, Felix Kolibay, Nora Schorscher, Georg Ertl, Ralf-Ingo Ernestus, Ulrich Vogel, Axel Franke, Barbara Kowalzik

**Affiliations:** 1 German Society of Hospital Disaster Response Planning and Crisis Management, DAKEP e.V., Cologne, Germany; 2 Section Emergency- and Disaster Relief Medicine, Department of Anaesthesia and Critical Care, University Hospital of Wuerzburg, Oberduerrbacherstrasse 6, 97080 Wuerzburg, Germany; 3 Staff Unit Hospital Alarm and Emergency Planning and Crisis Management, Hospitals of the City of Cologne, Cologne, Germany; 4 Staff Department Clinical Affairs and Crisis Management of the Medical Director, University Hospital of Cologne, Cologne, Germany; 5 Board of Directors, University Hospital of Wuerzburg, Wuerzburg, Germany; 6 Institute for Hygiene and Microbiology, Infection Control Team, University of Wuerzburg and University Hospital of Wuerzburg, Wuerzburg, Germany; 7 Department of Trauma Surgery and Orthopaedics, Reconstructive Surgery, Hand Surgery and Burn Medicine, German Armed Forces Central Hospital of Koblenz, Koblenz, Germany; 8 Federal Office for Civil Protection and Disaster Assistance, Bonn, Germany

**Keywords:** Mass critical care, Disaster response, SARS-CoV-2, Hospital emergency plan

## Abstract

Mass critical care caused by the severe acute respiratory syndrome corona virus 2 pandemic poses an extreme challenge to hospitals. The primary goal of hospital disaster preparedness and response is to maintain conventional or contingency care for as long as possible. Crisis care must be delayed as long as possible by appropriate measures. Increasing the intensive care unit (ICU) capacities is essential. In order to adjust surge capacity, the reduction of planned, elective patient care is an adequate response. However, this involves numerous problems that must be solved with a sense of proportion. This paper summarises preparedness and response measures recommended to acute care hospitals.

## Background

Mass critical care is the predominant problem of the severe acute respiratory syndrome corona virus 2 (SARS-CoV-2) pandemic. It has led to a dramatic strain on intensive care in many countries around the world [1]. The situation was aggravated by a blatant lack of staff and essential supplies. Preparation and hospital emergency planning are crucial factors in order to successfully cope such a challenging situation.

Hospitals play an essential role in the response to the SARS-CoV-2 pandemic. On the basis of experience gained in individual hospitals, the German Society of Hospital Disaster Response Planning and Crisis Management (DAKEP) has developed comprehensive recommendations for the hospital management of the SARS-CoV-2 pandemic. These recommendations are summarised in this paper and became part of the German manual entitled “Hospital Operational Planning and Crisis Management”, which was recently released by the Federal Office for Civil Protection and Disaster Assistance [2, 3].

### Objectives of the pandemic planning in hospitals

The first priority is the definition of the aim to be pursued and achieved. In the event of a mass influx of critically ill patients defined as “Mass Critical Care”, the primary goal must be to maintain the response category “conventional care” or at least “contingency care” for as long as possible, taking into account current and accepted medical standards [4, 5]. “Crisis care” based on disaster medicine principles must be avoided at all costs or delayed as long as possible by appropriate measures [4, 5]. The three components, i.e. staff, space and supplies, are the essential interacting variables for the planning of care. Their availability and organisation determine the level of care that the affected hospital can provide [4, 5].

### Command and control

#### Command organisation

For a comprehensive management of a pandemic and mass critical care, hospitals have to switch from the conventional mode of leadership to crisis mode with appropriate incident command structures [6].

A hospital incident command (HIC) should contain the following staff sections:

- Staff management and administration (S1)
- Situational report (information gathering and assessment) (S2)
- Operational command (S3)
- Technology and logistics (S4)
- Communication, media and press (S5)
- IT and mobile services (S6)

Each staff section is managed by a section chief. The incident commander is the head of the HIC and carries the overall mission responsibility.

The operational command section (S3) must include infectious disease and infection control experts as well as representatives of the emergency and intensive care department. Heightened importance is given to the section logistics and medical technologies (space and supplies). This increased relevance has its rationale in the context of a worldwide shortage of material supply, pharmaceutical products and impairment of logistics and transportation.

The HIC requires a command post that, because of the infectious disease threats, meets the requirements of physical distancing. Lecture halls, large function rooms, etc. can be suitable. The necessary technical equipment, including video conferencing tools must be provided.

#### Command process

The meetings of the HIC should follow a fixed scheduled scheme. The following agenda has proven feasible in crisis management [7]:

1. Situational report
2. Situational assessment
3. Problem identification and prioritisation of problems
4. Development of solutions and new work assignments
5. Monitoring of previous assignments and decisions regarding their degree of implementation (Review)

The situational report and gathering of information may be subdivided into several internal and external subunits.

Internal factors (e.g.):

- Medical situation (e.g. number of patients, dynamics, medical characteristics)
- Current treatment capacity for COVID-19 patients and non-COVID-19 patients
- Representation of the hospital’s functionality (staff, space, supplies)

External factors (e.g.):

- Epidemiological development of the pandemic
- Situation of other facilities in the region
- Detection of relevant hot spots of transmission
- Novel regulations by local, regional and national authorities
- Evaluation of recommendations and novel scientific evidence

All activities and decisions of the HIC must be documented in an operation diary and a workflow system.

#### Communication of HIC decisions

The frequency of decisions in a pandemic situation and the effects these decisions have on hospital staff is extraordinary. A solid communication structure to the individual departments and to all employees including all professional groups may be a problem but is extremely important.

General information should be distributed on a daily scale via intranet and e-mails. Information concerning individual departments or clinics should be communicated separately. For this purpose, the designation of a contact person in each department is essential.

The decisions of the HIC are binding for all employees of the company, including managers. Directors of departments and other managers should be briefed and involved in decision making on a regular basis by the HIC.

### Functionality of the hospital

An essential prerequisite for patient care while increasing COVID-19 treatment capacity is the solid maintenance of the functionality of the hospital [8]. In the context of a pandemic, several interacting factors need special consideration. The interdependency of the components staff, space and supplies in terms of functionality is clearly evident during all phases of the SARS-CoV-2 pandemic.

#### Supplies

The hospital’s functionality is directly limited by a strained material supply situation, which is caused by disturbed supply chains and increased competition between medical care facilities. This applies to personal protective equipment (PPE), disinfectants, respirators and their disposable material, and drugs. The unavailability of N95 respirators and disinfectants proved to be a critical trigger to drastically reduce the elective patient care in order to maintain functionality. A complete lack of protective equipment is an event that must be avoided at all costs [6, 9]. As hospitals usually work with a stockpile of equipment lasting roughly 14 days at normal demand, this scenario would be achieved within 2 weeks if there is a lack of supplies. There are currently signs of a blatant shortage of material supplies all over the world. Both the Centers of Disease Control and Prevention (CDC) and the Robert Koch Institute (RKI) have issued recommendations to cope with this situation [10, 11]. The CDC recommends a stepwise graded management with the described steps of a conventional care, contingency care and crisis care [10]. IT- solutions are required for the HIC to transparently monitor burn rate and range of supplies.

#### Staff and space—prevention of nosocomial transmissions

With respect to hospitals’ functionality, the prevention of nosocomial transmissions affecting staff and patients is critical. The consistent separation of suspected and proven cases and the strict adherence to infection control recommendations are paramount in order to achieve this [10].

In order to ensure the separation of SARS-CoV-2-positive patients from other patient routes, hospitals have to take extensive measures including setting up separate areas [10]:

- In the emergency room
- On normal wards
- Within intermediate care (IMC) and ICU
- In the delivery rooms
- In the operating theatres

All these measures require additional staff as well as careful interdisciplinary and interprofessional planning.

#### Infection control measures, personal protective equipment and training

Training of and tight adherence to infection control measures recommended by the national and international bodies is of utmost importance for the protection of hospital staff and to avoid nosocomial transmissions of COVID-19. Training should be conducted on site and especially focus on donning and doffing of PPE. Video training might be a valid alternative [12]; however, face-to-face instruction might enhance compliance [13]. Special care should be taken of in COVID-19 treatment wards, where complementary workforces need to be deployed (i.e. medical students).

### Hospitals treatment capacity

A quick and effective method for maintaining or increasing the treatment capacity for COVID-19 patients in the short term is the reduction of elective medical care. This measure rapidly releases personnel and supply capacities while emergency care and treatments can be maintained.

#### Critical decision making

The question of which treatments to postpone in a pandemic situation is difficult to answer. In particular, the approach to patients who are not formally classified as emergency patients, but need treatment to prevent further deterioration, faces the treating physicians with a dilemma [14, 15]. The situation is further aggravated by the fact that these patients often require the scarce resources of intensive care medicine. This overall constellation is a professional, ethical and potentially even juridical dilemma. Currently treated patients, those patients awaiting admission and the lack of staff and supplies are interdependent in a reciprocal decision-making.

#### Management of elective patient care

One tool to manage the elective patient care in a sensible manner is their categorisation by treatment urgency [15].

The following example of categorisation by weeks of acceptable postponement proved to work well during the first wave of the pandemic from March to May 2020:

- Category I: 0–2 weeks
- Category II: 2–4 weeks
- Category III: 4–12 weeks
- Category IV: > 12 weeks

**Fig. 1:**
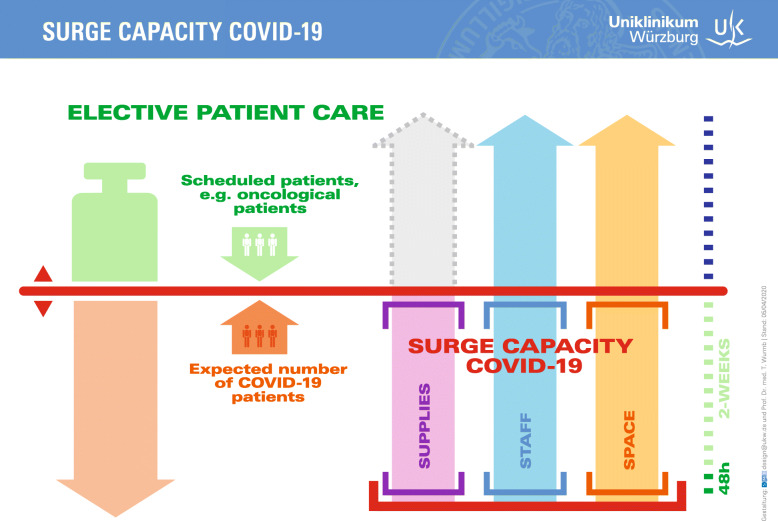
Managing surge capacity with regard to supplies, staff and space

The decision on “acceptable postponement” is in the responsibility of the treating consultant and should be consented in a board of consultants. It is recommended that on the basis of the situational reports, the HIC assesses daily, which categories can be released for treatment. In addition, daily interdisciplinary coordination of treatment indications must take place in order to prioritise the patients. An utmost degree of transparency of the individual disciplines is essential to prevent conflicts. This system allows the management of the elective patient care with an accuracy of approx. 48 h (Fig. 1).

#### Increasing the surge capacity for mass critical care

Appropriate measures must be taken to expand ICU capacities in order to achieve the abovementioned objective of maintaining the level of contingency care for as long as possible.

##### Material supply

Each additionally equipped intensive care bed requires, i.e. ventilators, disposable materials for ventilators, syringe pumps and monitors. Additional dialysis machines and consoles and equipment for extracorporal membrane oxygenation (ECMO) therapy are also required. In the case of contingency care, the supplemental material should be available in a reasonable time frame. Hick et al. describe availability within 12 h for the response level of contingency care [4, 5]. The material supply situation has proven to be particularly critical during the SARS-CoV-2 pandemic. If the burn rate exceeds purchase and stockpiles, this results in the transition to decompensated crisis care. Should a hospital find itself in such a shortage situation with crisis care, it is essential to proof and utilise regional, national or even international treatment capacities. This is especially true if triage to allocate scarce resources appears to be the last resort.

#### Staff

Even outside of the pandemic, the availability of trained nursing staff and doctors in intensive care is limited. In addition, there are staff shortfalls due to a lack of childcare facilities (in case of a lock down) as well as illness and quarantine due to SARS-CoV-2. Therefore, a short-term increase in qualified staff, which would be necessary to substantially increase intensive care capacities, is unrealistic. Accordingly, alternative concepts for recruitment must be considered early on.

Short-term measures to mobilise personnel for intensive care:

- Recruit and train nurses from other specialities
- Recruit and train doctors from other specialties
- Qualify nurses from regular ward to IMC and ICU
- Cooperate with other health-care institutions
- Recruit and train medical students.

It is of utmost importance that the level of contingency care is also maintained with regard to staff. If this is not possible in a short period of time due to high patient numbers, then care can only be provided at the cost of losing specialisation. This is a characteristic of crisis care [4, 5].

##### Organisation of intensive care capacities (space)

A major challenge is the organisation of intensive care capacities in a way that allows a step-by-step escalation of treatment capacities. Passing a “point of no return” must be as high up in the escalation scale as possible. An example of such a point of no return would be the opening of the operating areas for the ventilation therapy of COVID-19 patients [16]. Escalation possibilities to increase ICU capacities could be for example:

- IMC wards
- Post-surgical recovery rooms
- Areas of intervention (e.g. endoscopy)
- Operating theatre

The separation of COVID-19 from non-COVID intensive care units is to be strived for. Likewise, an operative non-COVID zone should be established and maintained under all circumstances in order to be able to adequately treat non-COVID emergencies. The CDC and the RKI give clear guidelines for this [10, 11].

The detailed structural framework and spatial planning must be tailored to the respective hospital and to the optimisation of infection control requirements (cohort isolation, no crossing paths, clear visual and spatial separation of areas). In order to achieve the goal of maintaining the response level of contingency care, rooms and areas which are—at least in their basic structure—equipped and intended for medical treatment, should be used as long as possible [4, 5]. Treatment in areas that do not meet these criteria, e.g. hotels, schools or function halls, marks the transition to the level of crisis care [4, 5].

### Lack of resources and mass critical care

At the level of crisis care, a hospital will reach the point where the lack of resources forces the treating physicians to triage patients and to allocate resources in a limited and prioritised manner. The distribution should be made in such a way that the greatest possible number of lives can be saved. Urgency and prospects of treatment success are important and critically discussed criteria for making this difficult decision [17–19]. End of life decisions are part of a doctor’s everyday medical experience. Under normal circumstances, however, these decisions are made in relation to the individual patient, his current prognosis, the treatment indication and the patient’s will. To consider a lack of resources, it requires starting a consensus-based approach for triage early on. Triage depends on severity of the deficiency situation and has therefore to be adapted promptly on a daily or even hourly basis. Individual triage warrants an experienced team of doctors and nurses in a joint effort guided by stringent criteria [17, 20–22]. Therefore, comprehensive triage concepts must be elaborated.

## Conclusion

The SARS-CoV-2 pandemic has drawn general attention to limited capacities and preparedness of hospitals and health care systems. Online available disaster response plans had to be deployed in order to maintain conventional care or at least to maintain contingency care. The procedures described here allow to avoid or delay crisis care by appropriate measures. National and local pandemic planning including a hospital incident command system are major components of preparedness of the national healthcare system.

## Data Availability

Not applicable

## Abbreviations

COVID-19: Corona virus disease
DAKEP: German Society of Hospital Disaster Response Planning and Crisis Management
ECMO: Extracorporal membrane oxygenation
PPE: Personal protective equipment
HIC: Hospital incident command
IMC: Intermediate care unit
ICU: Intensive care unit
SARS-CoV-2: Severe acute respiratory syndrome corona virus 2
